# Aggregation and Colloidal Stability of Commercially Available Al_2_O_3_ Nanoparticles in Aqueous Environments

**DOI:** 10.3390/nano6050090

**Published:** 2016-05-13

**Authors:** Julie Mui, Jennifer Ngo, Bojeong Kim

**Affiliations:** Department of Earth and Environmental Science, College of Science and Technology, Temple University, Philadelphia, PA 19122, USA; julie.mui@temple.edu (J.M.); jennifer.ngo@temple.edu (J.N.)

**Keywords:** aluminum oxide, nanoparticles, humic acid, montmorillonite, aggregation

## Abstract

The aggregation and colloidal stability of three, commercially-available, gamma-aluminum oxide nanoparticles (γ-Al_2_O_3_ NPs) (nominally 5, 10, and 20–30 nm) were systematically examined as a function of pH, ionic strength, humic acid (HA) or clay minerals (e.g., montmorillonite) concentration using dynamic light scattering and transmission electron microscopy techniques. NPs possess pH-dependent surface charges, with a point of zero charge (PZC) of pH 7.5 to 8. When pH < PZC, γ-Al_2_O_3_ NPs are colloidally stable up to 100 mM NaCl and 30 mM CaCl_2_. However, significant aggregation of NPs is pronounced in both electrolytes at high ionic strength. In mixed systems, both HA and montmorillonite enhance NP colloidal stability through electrostatic interactions and steric hindrance when pH ≤ PZC, whereas their surface interactions are quite limited when pH > PZC. Even when pH approximates PZC, NPs became stable at a HA concentration of 1 mg·L^−1^. The magnitude of interactions and dominant sites of interaction (basal planes *versus* edge sites) are significantly dependent on pH because both NPs and montmorillonite have pH-dependent (conditional) surface charges. Thus, solution pH, ionic strength, and the presence of natural colloids greatly modify the surface conditions of commercial γ-Al_2_O_3_ NPs, affecting aggregation and colloidal stability significantly in the aqueous environment.

## 1. Introduction

Nanoparticles (NPs), one of the major classes of nanomaterials, define the particle size class in the range of 1 to 100 nm. Due to their small size, NPs often possess novel physical and chemical properties in comparison to their bulk counterparts, such as higher sorption capacity, greater chemical reactivity, and useful optical signatures. Successful commercialization of their unique properties can be found in various fields, including electronics, cosmetics, biomedical and pharmaceutical sciences, energy technologies, catalytic and material applications, as well as environmental remediation [1,2,3,4,5,6]. Among the manufactured NPs, metal oxides NPs are in highest demand with one conservative estimate predicting growth in production up to 1.7 million metric tons by 2020 [7].

Aluminum oxide (Al_2_O_3_) NPs are ranked second in the total market production, accounting for approximately 20% of all nanomaterials manufactured and produced in 2012 [7,8,9]. They are extensively used for commercial products such as high performance ceramics, cosmetic fillers, packing materials, polishing materials, semiconductor materials, paints, composite materials and resins, wear-resistant reinforcement and advanced waterproof materials, catalyst, and catalyst carriers [10,11,12,13,14,15,16,17]. Especially, γ-Al_2_O_3_ NPs, one of the Al_2_O_3_ NP forms, are very important in high-technology products such as catalysts or catalyst support materials because of their small particle size, high surface area, and, therefore, enhanced catalytic activities. Currently, Al_2_O_3_ and titanium dioxide (TiO_2_) NPs are the only metal oxide NPs that can be fabricated in bulk through both bottom-up and top-down approaches; therefore, they are more economically favorable to use [18].

However, the extensive existing and continually growing uses of Al_2_O_3_ NPs in commercial applications has raised environmental concerns over potential adverse ecological effects. For instance, recent studies have reported the toxicity of Al_2_O_3_ NPs on various aquatic organisms including (micro) algae species [19,20,21], aquatic invertebrates [22,23], as well as zebra fish [24]. Furthermore, the toxic effects of Al_2_O_3_ NPs are also reported on the model organisms for ecotoxicity tests, such as *Bacillus subtillis*, *Escherichia coli*, *Pseudomonas fluorescens* [25,26], and *Caenorhabditis elegans* [27], as well as on mammalian cell lines [28,29].

Despite growing number of ecotoxicity studies, very few studies have investigated the environmental behavior of Al_2_O_3_ NPs in the natural aquatic settings [30]. Understanding the aggregation and colloidal stability of manufactured Al_2_O_3_ NPs in natural waters is essential to accurately evaluate their toxicity on aquatic organisms. Most likely, abiotic factors such as pH, ionic strength, and the presence of natural colloids (natural organic matter (NOM) and clay minerals) will have a significant impact on the aggregation phenomena, and therefore, influence the ability of Al_2_O_3_ NPs to form stable dispersions in natural waters.

In particular, the roles of humic acid (HA) and clay minerals on the aggregation chemistry of Al_2_O_3_ NPs are of importance in natural water systems because these natural colloids are ubiquitous and present in much higher concentration than manufactured NPs in the environment [31]. Studies with NPs of TiO_2_ [32,33,34], zinc oxide [35], and magnetite [36,37] have shown that HA can significantly enhance NP stability and mobility through sorption on the mineral oxide surfaces. Upon coating the surfaces, aliphaticity and polarity of HA further affect the colloidal stability of Al_2_O_3_ NPs [38]. Smectite clays, such as montmorillonite, are 2:1 layered aluminosilicates, and have permanent negative (−) structural charges [39,40]. In addition, unique properties of clay minerals, such as swelling, intercalation, and ion-exchange, add complexity and heterogeneity in surface interactions with manufactured NPs, further affecting their fate and behavior in the environment [41]. Ultimately, all these surface interactions will impact the entry or uptake mode of Al_2_O_3_ NPs by aquatic organisms in the environment, and therefore, need to be systematically investigated.

The primary objective of this study was therefore to investigate the aggregation and stability phenomenon of commercially available γ-Al_2_O_3_ NPs as a function of the solution pH, ionic strength (with common monovalent and divalent salts, NaCl and CaCl_2_), as well as the presence of natural organic matter and clay minerals for better understanding of the colloidal behavior of Al_2_O_3_ NPs in aqueous environments. In this study, we characterized and tested three nominally different sizes of commercially available γ-Al_2_O_3_ NPs, including 5 nm, 10 nm, and 20–30 nm. Montmorillonite (a natural, swelling-smectite clay) and HA were used as model clay and model natural organic acid, respectively. In mixed systems with montmorillonite or HA, we chose to study three pH regions aligned with the point of zero charge (PZC) of Al_2_O_3_ NPs: pH = 5 (pH < PZC); pH = 7.5–8 (pH = PZC); and pH 10 (pH > PZC) to further investigate the pH-dependence on the NPs aggregation and stability in the presence of clay minerals or HA. The observed NPs aggregation in those mixed systems was then compared to aggregation in the systems without HA (or clay). We employed X-ray diffraction (XRD), transmission electron microscopy (TEM), dynamic light scattering (DLS) and inductively-coupled plasma optical emission spectrometry (ICP-OES) for the detailed material characterization and measurements. Our investigation suggests γ-Al_2_O_3_ NPs remain stable as small aggregates in most natural water conditions, except when solution pH is close to PZC or has high ionic strength. Even when pH approximates PZC, NPs became stable in the presence of HA or montmorillonite through surface modification. TEM images illustrate the dynamic natures of surface interactions between γ-Al_2_O_3_ NPs and natural colloids in aqueous environments.

## 2. Results and Discussion

### 2.1. Material Characterization

**Figure 1:**
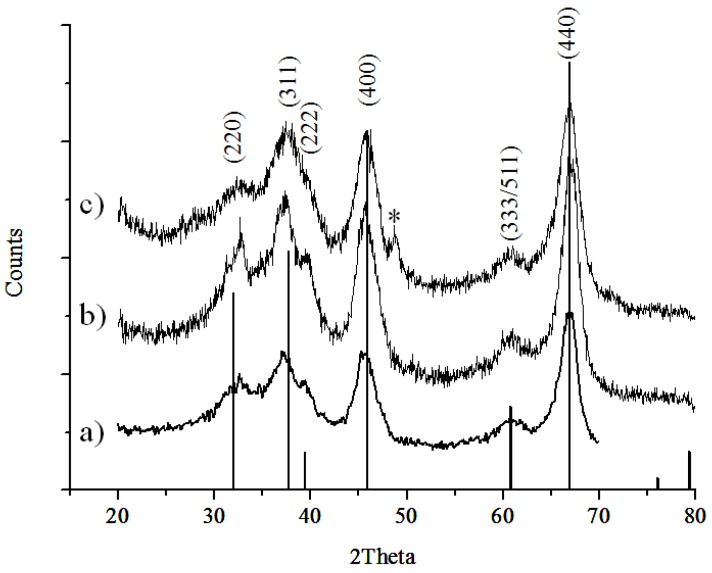
X-ray diffraction (XRD) patterns of commercial γ-Al_2_O_3_ nanoparticles (NPs) in size of: (**a**) 5 nm; (**b**) 10 nm; and (**c**) 20–30 nm. Note that 20–30 nm γ-Al_2_O_3_ NPs have a trace amount of boehmite (γ-AlOOH) (PDF 04-010-5683) marked with *, as an impurity.

Bulk crystal structure of the 5 nm, 10 nm, and 20–30 nm γ-Al_2_O_3_ NPs was determined using powder XRD analysis. The diffractograms of all γ-Al_2_O_3_ NPs samples display distinct diffraction peaks for γ-Al_2_O_3_ (ICDD JCPDS No. 98-000-0059; a spinel-type structure; space group Fd3m) [42,43,44], with all γ-Al_2_O_3_ NPs showing similarity in relative peak intensities (Figure 1). A trace amount of boehmite (AlOOH) (ICDD PDF 04-010-5683) was also detected for the 20–30 nm γ-Al_2_O_3_ (marked with asterisk in Figure 1c), indicating this particular sample of Al_2_O_3_ possesses mixtures of both minerals.

**Figure 2:**
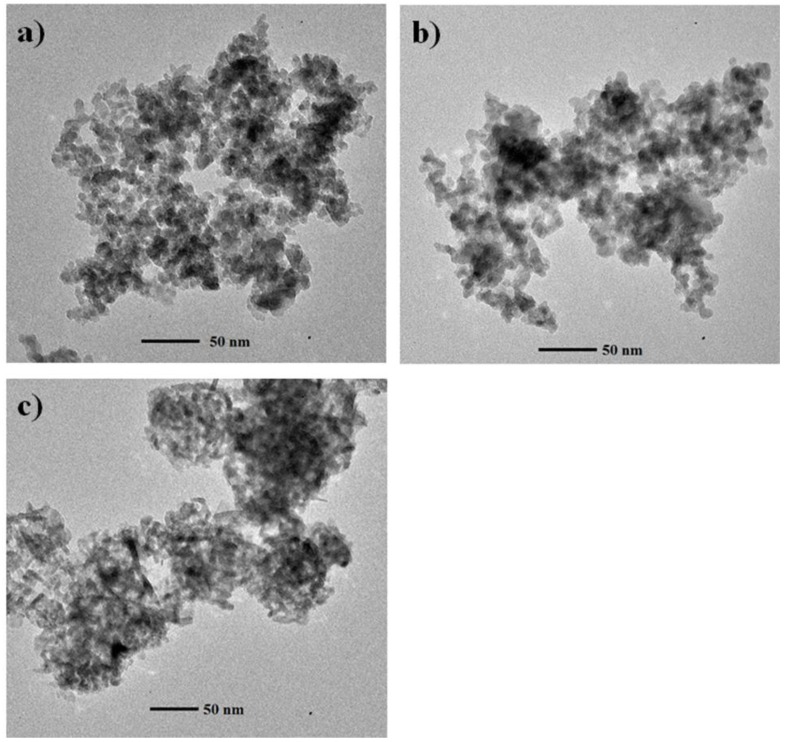
Bright field transmission electron microscopy (TEM) images of γ-Al_2_O_3_ NPs in size of: (**a**) 5 nm; (**b**) 10 nm; and (**c**) 20–30 nm.

TEM was used to study the primary particle size and morphology of the γ-Al_2_O_3_ NPs. Bright field (BF) TEM images of the 5 nm, 10 nm, and 20–30 nm γ-Al_2_O_3_ NPs are given in Figure 2a–c. Average primary particle sizes for the 5 nm and 10 nm γ-Al_2_O_3_ NPs are 10.1 nm (±2.51) (total number of 762 NPs on three different TEM grids individually analyzed) and 10.8 nm (±2.38) (total number of 367 NPs on two different TEM grids), respectively. Both 5 and 10 nm γ-Al_2_O_3_ NPs have a (pseudo)spherical shape. In contrast, the 20–30 nm γ-Al_2_O_3_ NPs have a mixture of spherical and rod shapes, where spherical and rod NPs have an average primary particle size of 7.18 (±2.43) (total number of 200 NPs on two different TEM grids) and 13.5 nm (±5.52) (rod shapes count 24.5% of the total NPs identified), respectively. The 20–30 nm γ-Al_2_O_3_ NPs are heterogeneous in size, morphology, and structure when compared to the 5 and 10 nm γ-Al_2_O_3_ NPs.

Particle size, morphology and structure are often the most influential factors to control the environmental fate and behavior of NPs [45,46,47]. As shown, 5 and 10 nm γ-Al_2_O_3_ NPs possess quite similar physicochemical characteristics in size, morphology, and structure, although the manufacturers claim that the particles are different in size. The primary particle size of the 20–30 nm γ-Al_2_O_3_ NPs was measured to be much smaller than the 20–30 nm. Further, both XRD and TEM analyses agreed that the 20–30 nm γ-Al_2_O_3_ NPs were heterogeneous in terms of structure and particle morphology. Manufactured γ-Al_2_O_3_ NPs are produced by incinerating amorphous or boehmite precursors at temperatures between 350 and 400 °C [42]. However, incomplete incineration during the manufacturing process may result in incomplete transformation and yield mixtures of precursors and NPs in the final product. Thus, caution is strongly advised when using commercially available NPs for ecotoxicity studies without detailed size, morphology, and structure analysis. Although the results of the material characterization disagree with the manufacturer’s information about the average size of the commercial NPs, we named the 5, 10, and 20–30 nm γ-Al_2_O_3_ NPs in the present work based upon the size ranges claimed by manufacturers.

### 2.2. Influence of Solution pH on Aggregation of Al_2_O_3_ NPs

**Figure 3:**
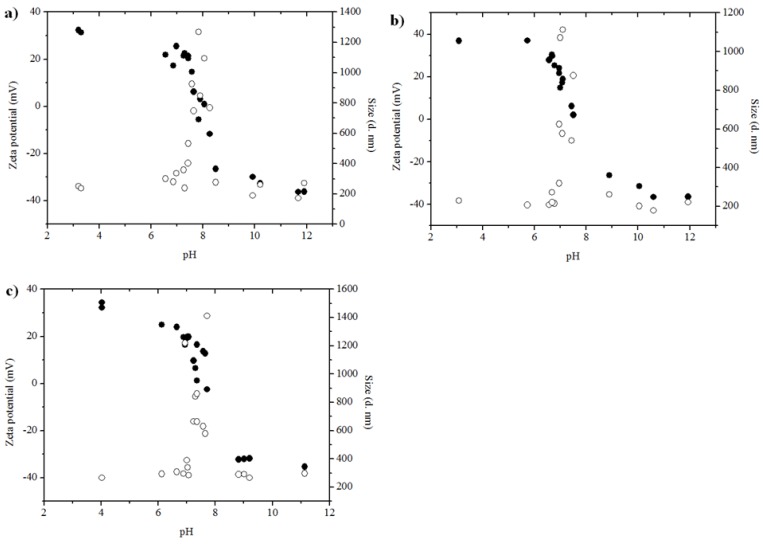
Z-average (Z_ave_) hydrodynamic diameter (○) and ζ potential (●) of: (**a**) 5 nm; (**b**) 10 nm; and (**c**) 20–30 nm γ-Al_2_O_3_ NP aggregates, as a function of pH.

The surface charge (ζ potential) and z-average (Z_ave_) hydrodynamic diameter of γ-Al_2_O_3_ NP aggregates were measured as a function of solution pH (ranging from pH 3 to pH 12) in a dilute NaCl solution (1 mM NaCl). The relationship between ζ potential and Z_ave_ of γ-Al_2_O_3_ NP aggregates in solution was explored by plotting the measurements (without standard deviation) collected over the range of tested pHs (Figure 3).

All the γ-Al_2_O_3_ NPs show positive ζ potential values of 31.3–37.0 mV in acidic solution pH. The ζ potential decreased with increasing pH of the solution and reached the point of zero charge (PZC) at pH 7.5 to pH 8, which is in a good agreement with the literature [30]. By increasing the pH further, ζ potential becomes negative and remains negative at −35.3 to −36.5 mV through pH 12.

The Z_ave_ diameter of γ-Al_2_O_3_ NP aggregates was in the size range of 202 to 266 nm (polydispersity index (PDI), 0.199–0.210) in acidic solutions (pH < PZC). Then, a rapid increase in Z_ave_ diameter of γ-Al_2_O_3_ NP aggregates was observed near the PZC, reaching a maximum value of 1113 to 1412 nm (PDI, 0.171–0.232). This reflects the enhanced aggregation of γ-Al_2_O_3_ NPs as a result of surface charge neutralization near the PZC. BF TEM images of 10 nm γ-Al_2_O_3_ NPs at PZC are provided in Figure S1, as a representative aggregate of all three γ-Al_2_O_3_ NPs studied in this work. The images show large aggregates of Al_2_O_3_ NPs (1020 to 1180 nm) with a very dense aggregate structure at PZC. Then, in basic solutions (pH > PZC), the Z_ave_ diameter of γ-Al_2_O_3_ NP aggregates ranged from 172 to 297 nm (PDI, 0.134–0.395), displaying similar size ranges as for acidic solutions.

All γ-Al_2_O_3_ NPs present very similar trends of ζ potential and Z_ave_ diameter as a function of pH, *i.e.*, there was no apparent size-dependent aggregation behavior of γ-Al_2_O_3_ NPs in pH titration curves. This is not surprising because XRD and TEM characterization indicated that the 5 nm and 10 nm γ-Al_2_O_3_ NPs do not have significantly different particle size and morphologies. Therefore, γ-Al_2_O_3_ NPs are either positively or negatively charged, and remain stable as small aggregates in solution except for a narrow pH region near the PZC (pH 7.5–8).

### 2.3. Influence of Ionic Strength on Aggregation of Al_2_O_3_ NPs

Two electrolyte solutions, NaCl and CaCl_2_, were used to examine the effect of ionic strength on γ-Al_2_O_3_ NP aggregation through DLS measurements. The same γ-Al_2_O_3_ NP concentration (614 mg·L^−1^) was used in all tests with NaCl or CaCl_2_ concentration ranges of 1 to 1000 mM. Typical Na^+^ concentrations in natural waters range from 43 μM (e.g., rain and river water) to 485 mM (e.g., seawater) and typical Ca^2+^ concentrations range from 25 μM to 11 mM, respectively. Therefore, the concentration ranges tested in this study suitably cover natural water conditions [48]. The pH of the electrolyte solutions was checked regularly to make sure the pH was consistent and much lower (5.7–6.2) than the PZC of the γ-Al_2_O_3_ NPs.

**Figure 4:**
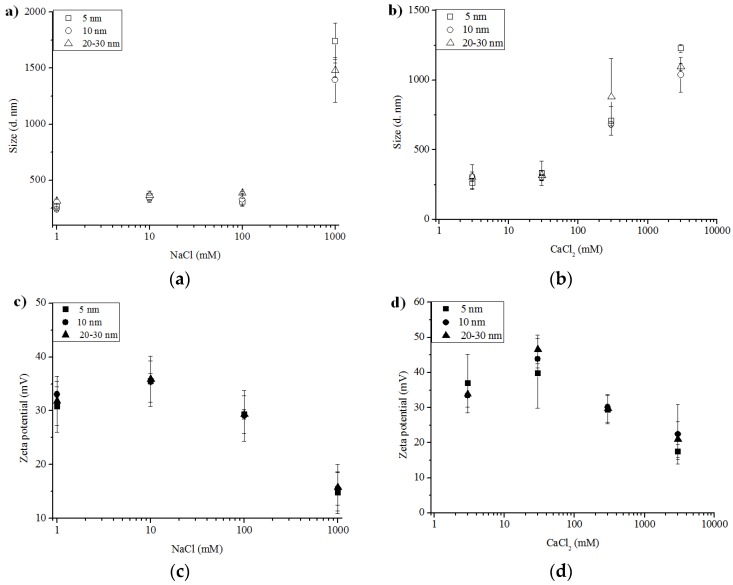
(**a**) Z_ave_ hydrodynamic diameter of 5 nm (□), 10 nm (○), and 20–30 nm (△) γ-Al_2_O_3_ NP aggregates, as a function of ionic strength (NaCl); (**b**) Z_ave_ hydrodynamic diameter of 5 nm (□), 10 nm (○), and 20–30 nm (△) γ-Al_2_O_3_ NP aggregates, as a function of ionic strength (CaCl_2_); (**c**) ζ potential of 5 nm (■), 10 nm (●), and 20–30 nm (▲) γ-Al_2_O_3_ NP aggregates, as a function of ionic strength (NaCl); and (**d**) ζ potential of 5 nm (■), 10 nm (●), and 20–30 nm (▲) γ-Al_2_O_3_ NP aggregates, as a function of ionic strength (CaCl_2_).

Figure 4 presents Z_ave_ measurements of γ-Al_2_O_3_ NPs as a function of solution ionic strength. The concentration ranges are equivalent to solution ionic strength of NaCl from 1 to 1000 mM, and of CaCl_2_ from 3 to 3000 mM. As shown, Z_ave_ measurements of γ-Al_2_O_3_ NPs do not show significant aggregation at low and medium ionic strength conditions. However, a significant increase in the Z_ave_ diameter was evident at 1000 mM for NaCl (the highest NaCl treatment in this study) (Figure 4a) and beginning at 300 mM with an increasing aggregation size through 3000 mM for CaCl_2_ (Figure 4b). The size distribution increased (*i.e.*, increasing standard deviation) as a function of increasing ionic strength for all the γ-Al_2_O_3_ NPs. The 5 nm γ-Al_2_O_3_ NPs had the largest Z_ave_ among all γ-Al_2_O_3_ NPs at 1000 mM of NaCl or 3000 mM of CaCl_2_.

Positive ζ potential values were measured for all the γ-Al_2_O_3_ NPs regardless of the solution type and ionic strength, but ζ potential values were significantly lower at the highest ionic strength (Figure 4c,d). The decrease in ζ potential is congruent with an increase in Z_ave_ diameter of γ-Al_2_O_3_ NP aggregates at higher ionic strength. All the γ-Al_2_O_3_ NPs present very similar trends in both ζ potential and size measurements. In general, increasing the ionic strength of the electrolyte solution compresses the diffused layer associated with the NPs, resulting in lowered ζ potential on the surfaces and diminished repulsions between NPs, and therefore, promoting particle aggregation. Similar observation was also made for several metal oxide NPs, including γ-Al_2_O_3_ [49], magnetite (Fe_3_O_4_) [36], TiO_2_ [50,51], and cerium oxide (CeO_2_) [52]. Thus, the presence of Na^+^ and Ca^2+^ will have a significant impact on the particle surface charges and thereby, particle aggregation, in natural waters where pH is lower than PZC.

### 2.4. Influence of Humic Acid on Aggregation of Al_2_O_3_ NPs

The effect of humic acid (HA) concentration (1, 5, 10, 20, and 50 mg·L^−1^) on the aggregation and stabilization of 10 nm γ-Al_2_O_3_ NPs was studied under three different pHs: pH < PZC; pH = PZC; and pH > PZC. 10 nm γ-Al_2_O_3_ NPs were chosen because their size, morphology, and structure were well defined and uniform such that the treatment effects could be attributed to solution pH and HA concentration but not variation in NP properties. In the absence of HA, 10 nm γ-Al_2_O_3_ NPs form large aggregates up to 1113 nm at PZC (Figure S1).

**Figure 5:**
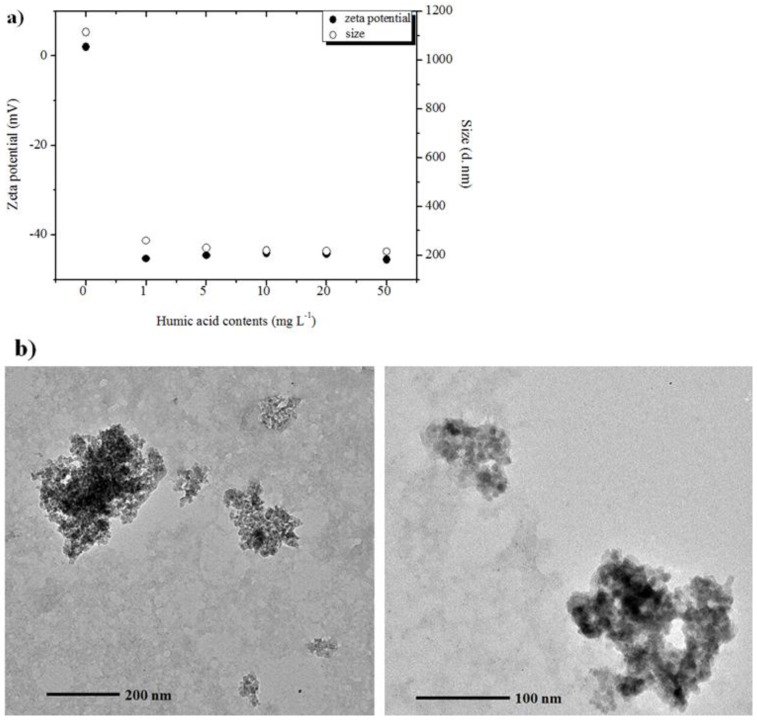
(**a**) Z_ave_ hydrodynamic diameter (○) and ζ potential (●) of 10 nm of γ-Al_2_O_3_ NPs as a function of HA concentration at pH close to the point of zero charge (PZC) of Al_2_O_3_; and (**b**) bright field TEM images of 10 nm γ-Al_2_O_3_ NPs with 10 mg·L^−1^ of HA at pH close to PZC of Al_2_O_3_.

HA possesses a negative surface charge (−28.0 to −49.0 mV) from pH 4 to 12, with a Z_ave_ diameter ranging from 198 to 283 nm (PDI = 0.369–0.474). In solution with pH at or below PZC, γ-Al_2_O_3_ NPs are positively charged or nearly neutral (~2 mV), whereas HA is negatively charged. All HA treatments resulted in a significant decrease in both the size and ζ potential of γ-Al_2_O_3_ NP aggregates, indicating dramatic changes in surface properties of the Al_2_O_3_ NPs in the presence of HA through electrostatic interaction. Figure 5a presents both the ζ potential and the size of γ-Al_2_O_3_ NP aggregates in the mixed system with HA at pH = PZC. HA likely increases NP stability through surface modification by electrostatic interactions as evidenced by a significant decreasing trend in both size and ζ measurements. This trend continues with increasing HA in the mixed system, although the trend appears to reach a plateau around 10 mg·L^−1^ of HA. Therefore, 10 nm, γ-Al_2_O_3_ NPs with 10 mg·L^−1^ of HA were chosen for imaging of the aggregate structures by TEM. A kinetic study with HA and Al_2_O_3_ reported different adsorption rates of HA on Al_2_O_3_ by solution pH [53]. For example, the adsorption of HA on Al_2_O_3_ surfaces readily occurs in low pH (~5) through electrostatic interactions. However, when solution pH increases, an electrostatic barrier develops between the negative surfaces of HA and Al_2_O_3_ and the sorption process is strongly inhibited, thereby significantly lowering the adsorption rate.

Representative BF TEM images are given in Figure 5b. In these images, small aggregates of γ-Al_2_O_3_ NPs show with dark contrast, while organic-rich HA matrix provides a lighter, patchy background. In the presence of HA, the sizes of γ-Al_2_O_3_ NP aggregates ranged from 75 to 358 nm at pH = PZC, which are much smaller than the sizes of NP aggregates at PZC in the absence of HA. In addition, γ-Al_2_O_3_ NP aggregates appeared to be isolated or compartmentalized by HA, which may prevent them from forming larger aggregates at that particular pH. Excess HA therefore enhances stability of Al_2_O_3_ NPs at PZC through combined effects of electrostatic interactions and steric hindrance. However, when pH is higher than PZC, both γ-Al_2_O_3_ NPs and HA are negatively charged and little interaction between them is expected. TEM images show the NPs and HA acting as separate identities in the suspension, suggesting very little interaction when pH is higher than PZC (Figure S2).

### 2.5. Influence of Montmorillonite on Aggregation of Al_2_O_3_ NPs

Montmorillonite, a swelling-smectite clay, possesses permanent negative charges on the basal planes due to isomorphic substitution of the Si and Al ions in the structure, as well as conditional charges at the amphoteric edge sites (mainly, Si-OH and Al-OH) [54,55]. The ζ potential measurements on montmorillonite in this study are also in line, presenting all negative values of −27.3 to −40.3 mV in solutions of pH 3.2 to 11, with a decreasing trend as pH increases (Figure S3). The PZC of the edge groups on montmorillonite is estimated at pH ~6.5 [40,56]. Therefore, dissociation of surface hydroxyl groups at edge sites is expected to occur when pH is higher than 6.5, contributing more net negative surface charges to the ζ potential measurement. BF TEM images of montmorillonite alone at pH 7.5 to 8 (*i.e.*, PZC of Al_2_O_3_ NPs) were provided in Figure S4. As shown, individual montmorillonite clay platelets have an irregular polygonal shape and display a combination of parallel stacked platelets and randomly oriented platelet patches. Individual clay platelets are only a few nanometers thick. However, once they form stacks and patches, clay platelets produce thicker sample regions for TEM analysis, which result in relatively dark contrast on those images [57]. It is also worth mentioning that dehydration of hydrated structures of clay minerals in the TEM vacuum or electron beam damage may occur during the TEM analysis, producing artifacts in the results [57].

**Figure 6:**
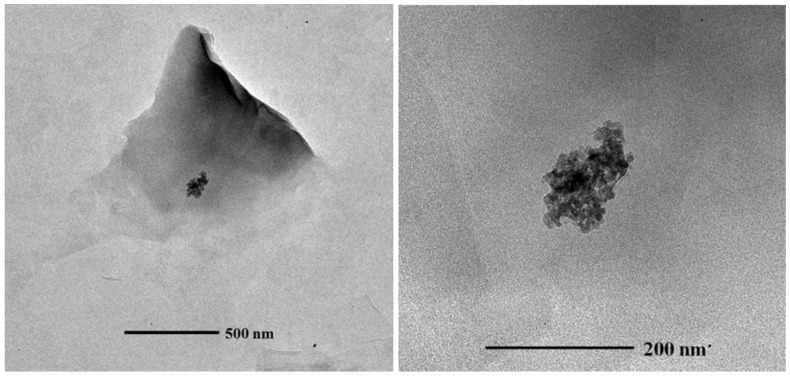
Bright field TEM images of 10 nm γ-Al_2_O_3_ NPs with montmorillonite at pH < PZC of Al_2_O_3_ NPs.

**Figure 7:**
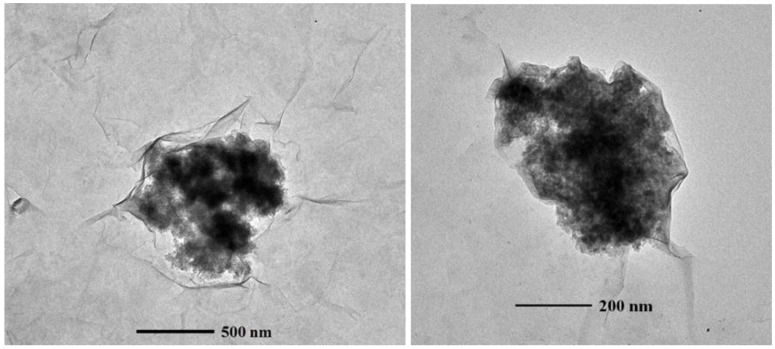
Bright field TEM images of 10 nm γ-Al_2_O_3_ NPs with montmorillonite at PZC of Al_2_O_3_ NPs.

**Figure 8:**
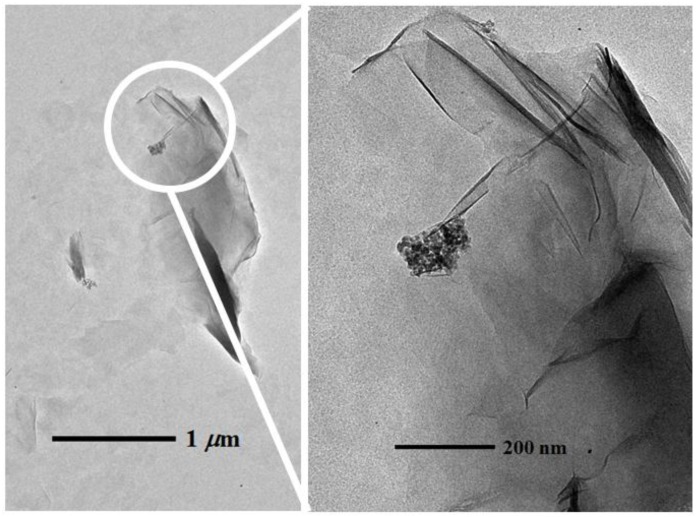
Bright field TEM images of 10 nm γ-Al_2_O_3_ NPs with montmorillonite at pH > PZC of Al_2_O_3_ NPs.

The influence of montmorillonite (at 0.05 Al_2_O_3_ NPs to clay ratio (*w*/*w*)) on the aggregation and stability of the 10 nm γ-Al_2_O_3_ NPs was studied in the mixed system under three different pHs: pH = 5.6 (pH < PZC of Al_2_O_3_ NPs); pH = 7.5 to 8 (pH = PZC of Al_2_O_3_ NPs); and pH = 9.0 (pH > PZC of Al_2_O_3_ NPs). The ratio of Al_2_O_3_ NPs to clay was chosen to simulate the clay minerals being dominated in aqueous environments (*i.e.*, clay minerals are present at much larger concentrations than the manufactured Al_2_O_3_ NPs in environmental settings) [31]. At low pH (<PZC of Al_2_O_3_ NPs), 10 nm γ-Al_2_O_3_ NPs are positively charged, the montmorillonite are negatively charged, and most of the negative surface charges are from their basal planes. The ζ potential measurement in the mixed system at low pH is negative (−35.2 ± 1.98 mV), indicating that montmorillonite has a significant interaction with positively-charged γ-Al_2_O_3_ NPs. Representative BF TEM images of montmorillonite with γ-Al_2_O_3_ NPs are given in Figure 6, where the interaction of the basal planes of montmorillonite with γ-Al_2_O_3_ NPs is evident. At PZC of Al_2_O_3_ NPs, both the basal planes and edges of montmorillonite are expected to be negatively charged. The ζ potential measurement in the mixed system is −34.7 (±0.07 mV). At PZC, γ-Al_2_O_3_ NPs is neutral or slightly positive (~2 mV). Figure 7 shows BF TEM images of montmorillonite with γ-Al_2_O_3_ NPs where the edge sites of montmorillonite are the dominant places of interaction with γ-Al_2_O_3_ NPs. At higher pHs (>PZC of Al_2_O_3_ NPs), both γ-Al_2_O_3_ NPs and montmorillonite are negatively charged, and therefore, the ζ potential measurement of the mixed system is also negative (−34.9 ± 0.57 mV). Similar to the HA system, very limited interaction of γ-Al_2_O_3_ NPs with montmorillonite was found in higher pH regions. Representative BF TEM images of montmorillonite with γ-Al_2_O_3_ NPs at/above PZC of Al_2_O_3_ NPs are given in Figure 8.

The role of basal planes and edge sites of montmorillonite clays seems important in the mixed system, especially at low pH (<6) or at PZC of Al_2_O_3_ NPs (pH 7.5 to 8), as Al_2_O_3_ NP aggregates appeared to be placed on the basal planes or surrounded by sheet edges or pockets of clay platelets on the TEM images. However, at high pH, their interaction is quite limited, where Al_2_O_3_ NP aggregates are found dangling at the clay edge sites on the TEM images.

## 3. Experimental Section

### 3.1. Materials

5 nm γ-Al_2_O_3_ NPs (99.99%) were purchased from US Research Nanomaterials, Inc. (Houston, TX, USA) in powder form. 10 nm (99.99%) and 20 to 30 nm (99.97%) were purchased from Nanostructured & Amorphous Materials, Inc. (Houston, TX, USA). All γ-Al_2_O_3_ NP stock solutions were made to achieve the target concentration of 614 mg·L^−1^ in 1 mM NaCl solution by using a probe sonicator (Sonic Dismembrator Model 100, Fisher Scientific Inc., Pittsburgh, PA, USA) for 30 min. 1 mM of NaCl served as a background, except for the experiments with the electrolyte solutions having varying ionic strength. For the ionic strength experiments, the γ-Al_2_O_3_ NPs stock solution was made in 1, 10, 100, and 1000 mM of NaCl or CaCl_2_ by sonication. A fresh stock NP suspension was made and used for each set of the experiments.

The HA stock solution was prepared by using 10 g·L^−1^ of HA, sodium salt (technical grade, CAS# 68131-04-4, Sigma-Aldrich Corp., Saint Louis, MO, USA) in 1 mM NaCl solution with stirring for 24 h before the day of the experiment. The HA stock solution was then filtered through a Whatman^®^ ashless filter paper (Grade 42) (Fisher Scientific Inc., Nazareth, PA, USA), and stored in the dark prior to its use. Without pH adjustment, the HA stock solution was approximately pH 10.0. For the mixed system with Al_2_O_3_ NPs, the HA stock solution was diluted in a systematic manner to reach the total organic carbon (TOC) concentrations of 1, 5, 10, 20 and 50 mg·L^−1^ at target pHs of 4, 8, and 10.

A clay suspension was prepared by allowing several grams of montmorillonite powder (Bentonite clay, 200-mesh, #460438, Ward’s Science, Rochester, NY, USA) to settle in a 1000 mL graduate cylinder for 8 h. After 8 h, the top 10 cm of supernatant that contains only the clay-sized (<2 μm) fraction of the original material was siphoned from the cylinder. For the mixed system with Al_2_O_3_ NPs, 0.05 weight to weight ratio (*w*/*w*) of Al_2_O_3_ to montmorillonite was chosen for the present study to closely simulate the most of natural settings (*i.e.*, clays being dominant by mass). Dilute hydrochloric acid and sodium hydroxide solutions were used to adjust the pH of the solutions. All the solutions were made with NANOpure^®^ water (18.2 MΩ·cm water with 1–5 ppb TOC) (Thermo Fisher Scientific Inc., Pittsburgh, PA, USA).

### 3.2. Powder XRD Measurements

The dry, powder samples of γ-Al_2_O_3_ NPs were analyzed using Rigaku D/max-B powder X-ray Diffractometer with Cu radiation (35 kV, 15 mA) (Rigaku Americas Corp., Allison Park, PA, USA). The diffractometer was set up to measure in step-scan mode over the 2θ range from 19° to 80° at 0.6° 2θ/min with a step size of 0.05°. A pitted glass sample holder was used without glue. A standard quartz and silicon sample were analyzed prior to experiment samples to ensure proper instrument calibration. Data processing and analysis were performed using the JADE 9.0 software package (Jade software Corp., Jacksonville, FL, USA).

XRD analysis on montmorillonite powder samples was done by a D2 PHASER diffractometer equipped with Cu radiation (30 kV, 10 mA) and a high speed linear detector (LYNXEYE) (Bruker AXS Inc, Madison, WI, USA). The diffractometer was set up to measure in step-scan mode over the 2θ range from 5° to 65° at 1.0 s/step with a step size of 0.02°. Phase identification was performed using the DIFFRAC.EVA software package (Bruker AXS Inc, Madison, WI, USA) in combination with the ICDD PDF database. The result showed the very broad reflection at low angles, which is indicative of montmorillonite (main phase, shown in blue on Figure S5), with its composition of Al_0.86_Fe_0.1_H Li_0.08_Mg_0.14_O_10_Si_3.9_. It also showed the presence of trace amounts of other minerals, including quartz, gypsum, calcite, rutile, and aragonite.

### 3.3. Zeta Potential and Size Distribution Measurements

A zetasizer Nano ZSP (Malvern Instruments, Worcestershire, UK) was used to determine the ζ potential values as well as the Z_ave_ diameters of the γ-Al_2_O_3_ NP aggregates, HA, montmorillonite, and mixtures. The ζ potential measurement has 20 sub-runs with a delay of 5 seconds between them, whereas the size distribution (Z_ave_) has 12 sub-runs. Each sample ran three times to obtain the average values of the ζ potential and the Z_ave_ measurements. Each experiment had duplicated samples.

For the single-component system (*i.e.*, Al_2_O_3_ NP suspension), the ζ potential and Z_ave_ measurements of the Al_2_O_3_ NP aggregates were recorded as a function of solution pH or ionic strength. For the mixed system with HA, the ζ potential and Z_ave_ diameters were measured as a function of TOC concentration at pH close to PZC of Al_2_O_3_ NPs. All the experiments were carried out at room temperature. Preliminary data suggested the mixed system (with HA or clay) reached an equilibrium state after a few hours of reaction time (with stirring) and maintained it over the next few days. For consistency, a 24-h equilibration time was allotted for both the single-component and mixed systems for the ζ potential and Z_ave_ measurements.

### 3.4. Ultrafiltration

The presence of ionic Al species (not a form of NPs) in γ-Al_2_O_3_ NP suspensions by different treatments was also examined. Specifically, a portion of the Al_2_O_3_ NP suspension was taken and centrifuged with 10 k MWCO Amicon filters (EMD Millipore Corp., Billerica, MA, USA) at 3000 rpm for 15 min at 20 °C. The collected filtrates were then acidified with 1% nitric acid for inductively coupled plasma-optical emission spectrometry (ICP-OES, the Spectro Arcos ICP-OES) analysis. The results show that measured ionic Al concentrations in the filtrates ranged from 18 to 33 ppb for the three γ-Al_2_O_3_ NPs, indicating that none of the experimental conditions used for the present study produced a significant concentration of dissolved Al in the solutions. Thus, both current sonication and acidic pH solution conditions did not cause the dissolution of γ-Al_2_O_3_ NPs.

### 3.5. TEM Analysis

A drop of the sample solution was placed onto a 400-mesh, carbon-film coated Cu grid (Electron Microscopy Sciences, Hatfield, PA, USA) and allowed to evaporate. The JEOL JEM-1400 TEM (JEOL USA, Inc., Peabody, MA, USA) was used in bright field (BF) mode at 120 kV for imaging of γ-Al_2_O_3_ NPs and their aggregates.

## 4. Conclusions

The behavior of three commercial γ-Al_2_O_3_ NPs in aqueous environments was studied in a systematic manner, covering a wide range of natural water conditions. First, γ-Al_2_O_3_ NPs possess pH-dependent surface charges, with the PZC around 7.5 to 8. If pH < PZC, then γ-Al_2_O_3_ NPs are positively charged, whereas if pH > PZC then γ-Al_2_O_3_ NPs are negatively charged. Dissolution of these three γ-Al_2_O_3_ NPs did not occur under the pH conditions tested in this study (pH 3 to pH 12). Second, at pH < PZC, significant γ-Al_2_O_3_ NPs aggregation occurs only when the electrolyte solutions have high ionic strength (e.g., 1 M NaCl, and 300 mM–3 M CaCl_2_). Third, both HA and montmorillonite enhance the stability of γ-Al_2_O_3_ NPs at and below PZC through combined effects of electrostatic interactions and steric hindrance. However, very little interaction of γ-Al_2_O_3_ NPs with HA or montmorillonite is observed when pH > PZC, where the compounds act as two separate, negatively charged species in suspensions. Both γ-Al_2_O_3_ NPs and montmorillonite have conditional charges, and therefore, the solution pH determines the degree of their surface interactions as well as the sorption site(s) of such interactions. In general, if pH < PZC, then basal planes of clay platelets are dominant sites for the surface interactions with NPs. If pH approximates PZC, then edge sites are the dominant surface interaction sites. Thus, solution pH, ionic strength, and the presence of natural organic and inorganic colloids greatly modify the surface conditions of commercial γ-Al_2_O_3_ NPs, affecting aggregation and colloidal stability significantly in the aqueous environment.

## Supplementary Materials

The following are available online at http://www.mdpi.com/2079-4991/6/5/90/s1.

## Abbreviations

The following abbreviations are used in this manuscript.

BF	Bright Field
DLS	Dynamic Light Scattering
HA	Humic Acid
ICP-OES	Inductively-Coupled Plasma Optical Emission Spectrometry
NPs	Nanoparticles
PZC	Point of Zero Charge
TEM	Transmission Electron Microscopy
XRD	X-ray diffraction
