# Supplementary material for: Aggregation and Colloidal Stability of Commercially Available Al2O3 Nanoparticles in Aqueous Environments
Source: Nanomaterials (Basel). 2016 May 13;6(5):90. doi: 10.3390/nano6050090 (PMC5302499; doi:10.3390/nano6050090)
Supplement: Supplementary file 1 [file nanomaterials-06-00090-s001.pdf]

Supplementary Materials: Aggregation and Colloidal Stability of Commercially Available Al_2O_3 Nanoparticles in Aqueous Environments

Julie Mui, Jennifer Ngo and Bojeong Kim *

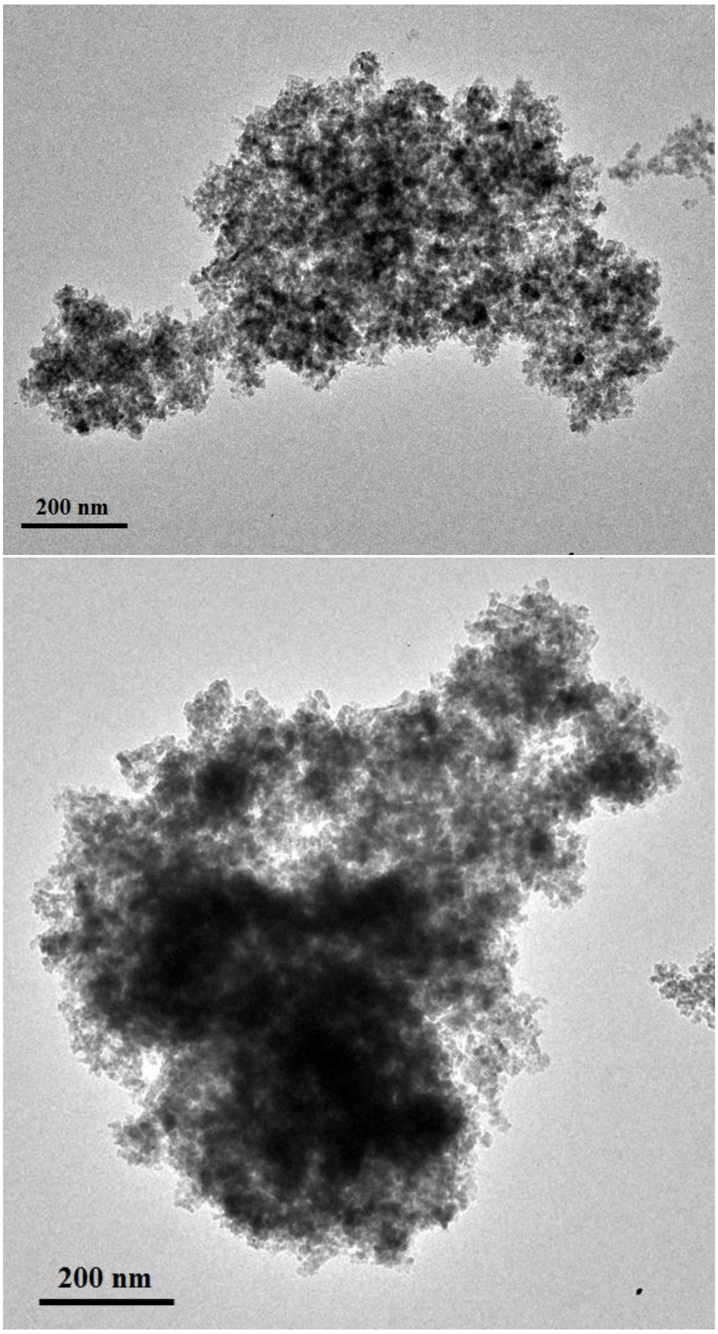

Figure S1. Bright field transmission electron microscopy (TEM) images of 10 nm γ-Al_2O_3 Nanoparticle (NP) aggregates at a point of zero charge (PZC).

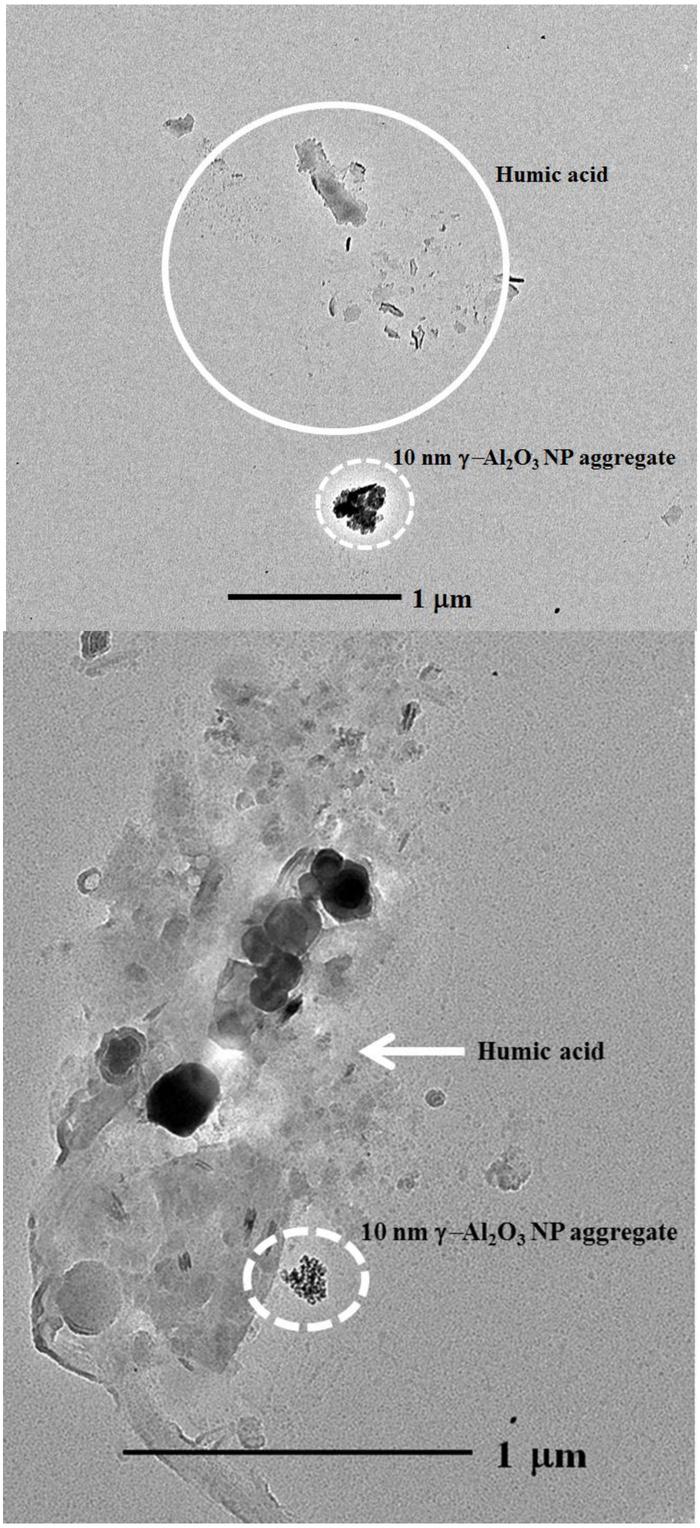

Figure S2. Bright field TEM images of 10 nm γ-Al_2O_3 NPs with 10 mg·L^{-1} of humic acid (HA) at pH > PZC. NP: Nanoparticle.

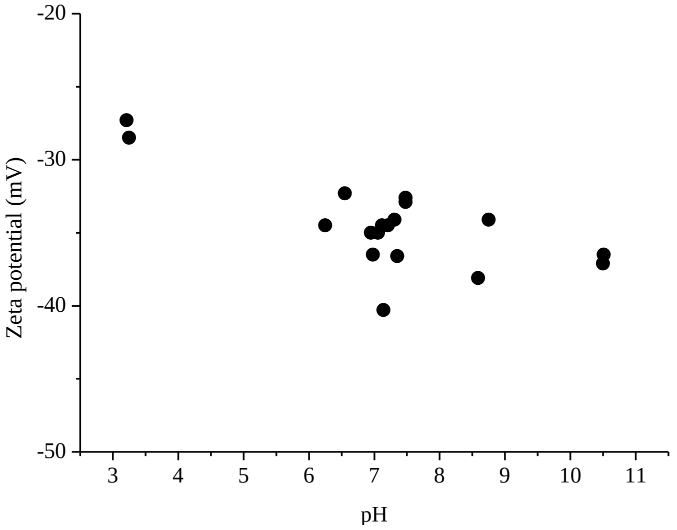

Figure S3. ζ potential measurements of montmorillonite, as a function of pH.

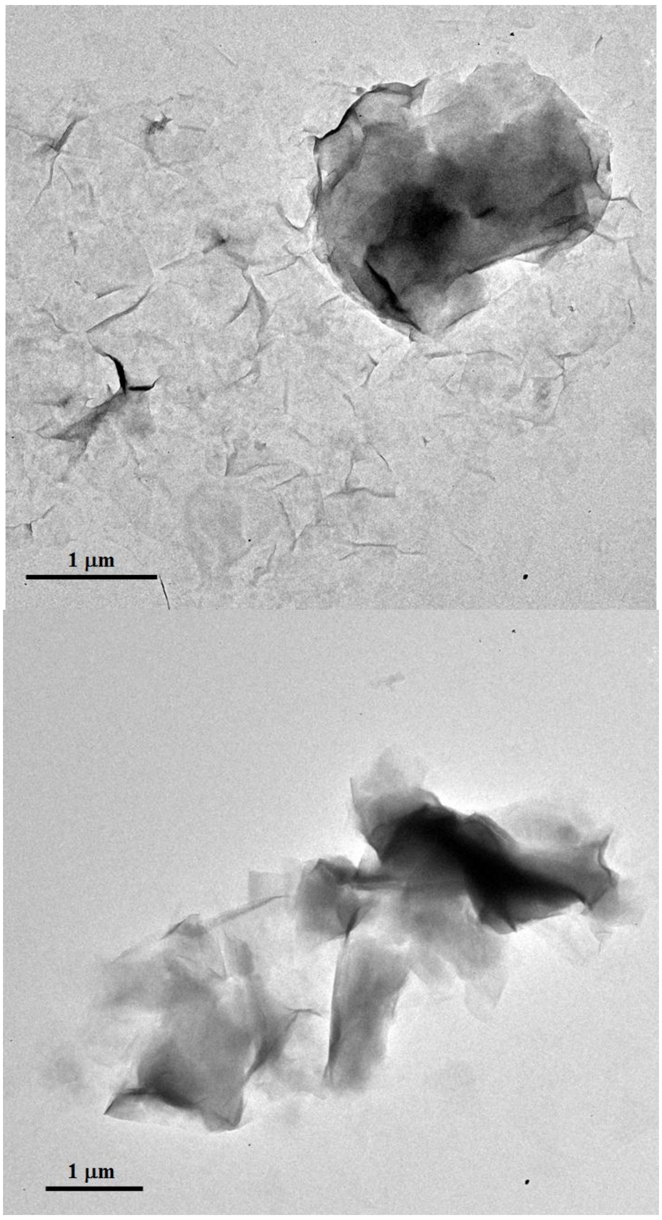

Figure S4. Bright field TEM images of montmorillonite at pH close to PZC of Al_2O_3 NPs.

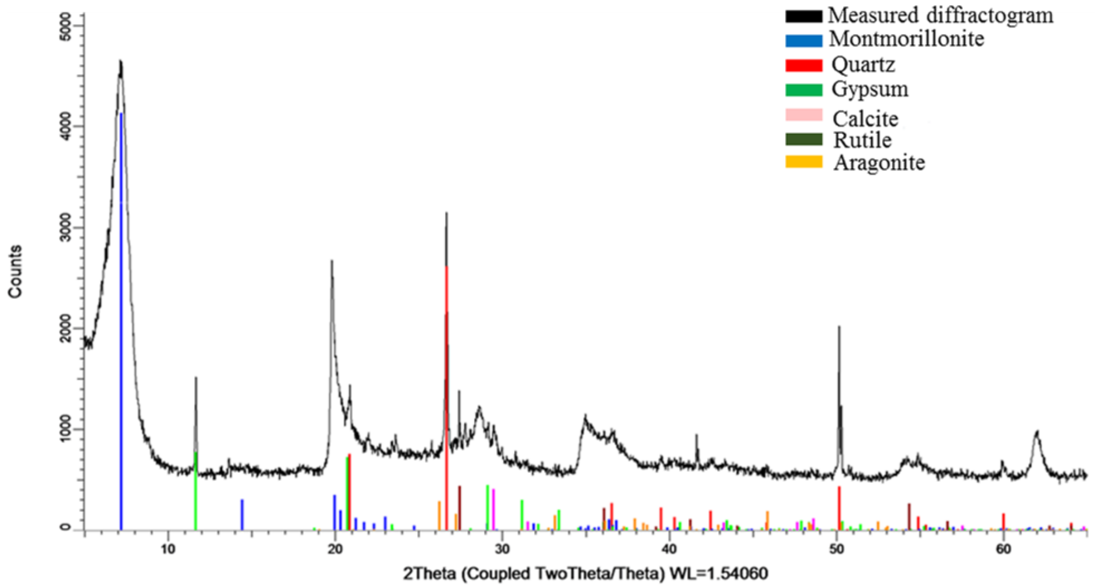

Figure S5. X-ray diffraction (XRD) pattern of montmorillonite. WL: Wavelength.

The X-ray diffraction (XRD) result showed the very broad reflection at low angles, which is indicative of montmorillonite (main phase, shown in blue), with its composition of $Al_{0.86}Fe_{0.1}H Li_{0.08}Mg_{0.14}O_{10}Si_{3.9}$. It also showed the presence of trace amounts of other minerals including quartz, gypsum, calcite, rutile, and aragonite (shown in red, green, pink, brown, and yellow, respectively).

© 2016 by the authors; licensee MDPI, Basel, Switzerland. This article is an open access article distributed under the terms and conditions of the Creative Commons by Attribution (CC-BY) license (http://creativecommons.org/licenses/by/4.0/).
